# Case report: Meningitis and intracranial aneurysm caused by mixed infection of oral microflora dominated by anaerobes

**DOI:** 10.3389/fneur.2022.889838

**Published:** 2022-08-05

**Authors:** Hongjiang Cheng, Lina Xu, Fengbing Yang, Longbin Jia, Doudou Zhao, Huimin Li, Wei Liu, Yujuan Li, Xiaoli Liu, Xia Geng, Jiaying Guo, Chen Ling, Jing Zhang

**Affiliations:** ^1^Department of Neurology, Jincheng People's Hospital Affiliated to Shanxi Medical University, Jincheng, China; ^2^Department of Rheumatology, Jincheng People's Hospital Affiliated to Shanxi Medical University, Jincheng, China; ^3^Graduate School of Changzhi Medical College, Changzhi, China

**Keywords:** oral microflora, anaerobes, meningitis, intracranial aneurysm, case report

## Abstract

**Introduction:**

Meningitis caused by oral anaerobic bacteria is rare, especially when complicated with an infected intracranial aneurysm. This paper has described an extremely rare case of bacterial meningitis caused by a mixed infection of oral microflora dominated by anaerobes, which developed cerebral infarcts, brain abscess, intracranial aneurysm, and severe hydrocephalus during treatment.

**Case report:**

We describe a 65-year-old male patient who was presented with fever and headache as the initial symptoms and then developed left ophthalmoplegia, right hemiplegia, and disturbance of consciousness. Brain imaging showed that intracranial lesions were increased progressively, and cerebral infarcts, brain abscesses, intracranial aneurysm, and severe hydrocephalus were appeared gradually. Eventually, we diagnosed it as anaerobic meningitis by making deoxyribonucleic acid sequencing from the brain abscess pus. After using an anti-microbial regimen that can sufficiently cover anaerobes, the patient's condition was effectively controlled.

**Conclusion:**

Anaerobic meningitis can cause a series of intracranial complications. Among them, the intracranial aneurysm is extremely rare. When evidence shows that the infection originates from oral flora, physicians should consider the possibility of this type of encephalitis. An early diagnosis and timely treatment are crucial to improving the prognosis.

## Introduction

The oral cavity harbors a diverse and abundant microflora. As part of the Human Microbiome Project, culture-independent molecular approaches have identified nearly 1,200 taxa of microbes in the human mouth (1). Under normal conditions, these organisms do not exhibit pathogenicity. However, in patients predisposed to infection or immunocompromised, it may act as an infectious pathogen to cause a series of diseases, such as intracranial invasion and serial intracranial complications (2, 3). The oral anaerobes, such as Prevotella, Fusobacterium, Bacteroides, and Peptostreptococcus, have been regarded as the opportunistic pathogen (3). Abscesses, bacteremia, and bone infections are the most common clinical presentation (2, 4). However, meningitis is rare, especially when complicated with an infected intracranial aneurysm. In this paper, we reported an extremely rare case of bacterial meningitis caused by a mixed infection of oral microflora dominated by anaerobes, which developed cerebral infarcts, brain abscess, intracranial aneurysm, and severe hydrocephalus during treatment.

## Case report

A 65-year-old male patient was admitted to the hospital because of fever and headache lasting for 5 days. The patient long had poor oral hygiene and 4 years ago, the patient underwent implantation of mandibular dentures. Fever usually occurred at noon and was generally under 38°C. In addition, the patient was presented with persistent heartburn, anorexia, asthenia, and night sweats. Blood routine examination revealed that the white blood cell (WBC) count was 12.08 × 10^9^/L (normal range, 4–10 × 10^9^/L) with neutrophil leukocytosis of 71.1%. No significant abnormality was found in the heart or lungs of the patient. Then the patient was admitted to the Department of Infectious Diseases.

**Figure 1:**
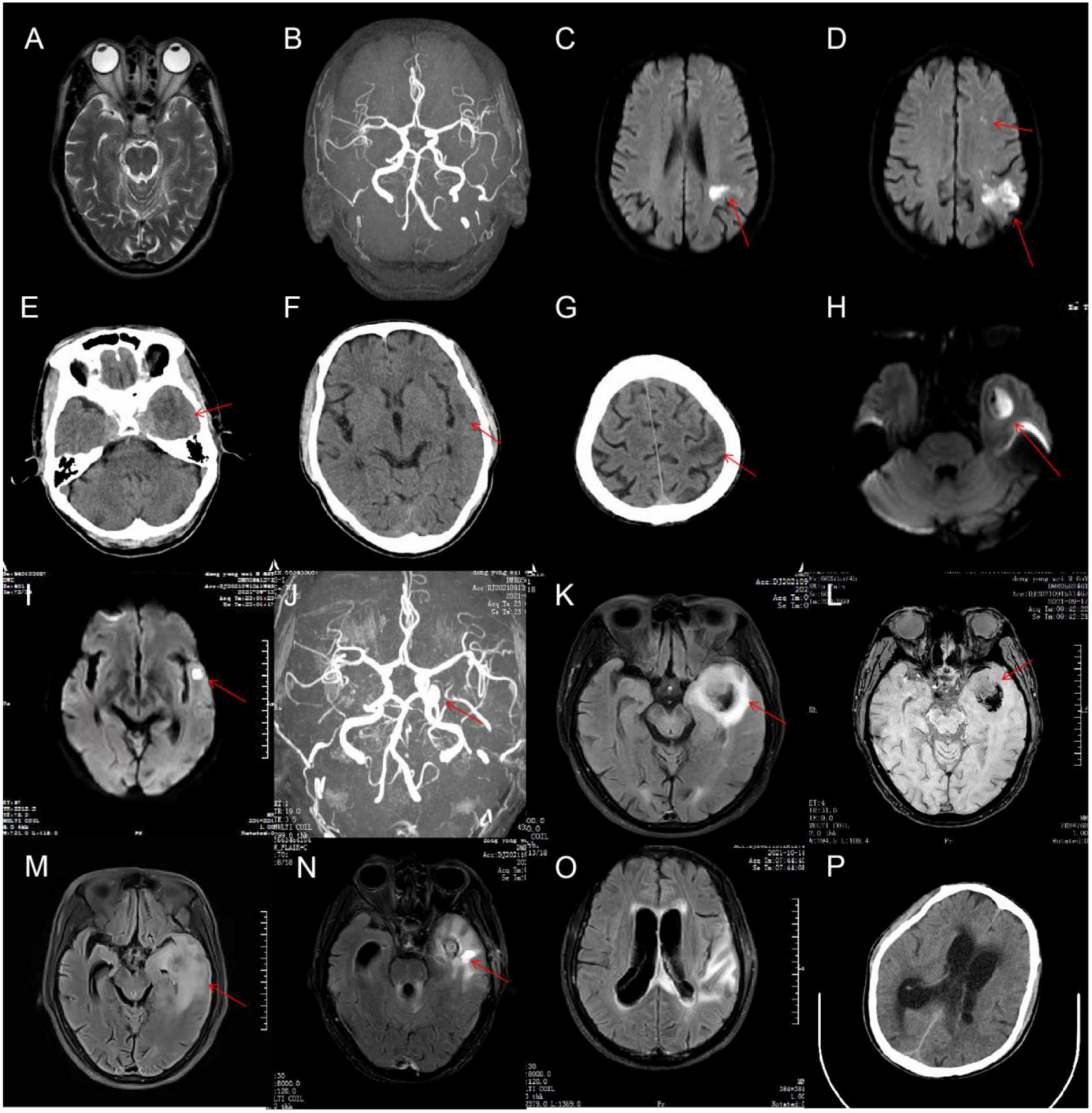
On admission, cranial magnetic resonance imaging (MRI) of the patient was normal **(A)**, and there was no evidence of intracranial aneurysm on brain magnetic resonance angiography (MRA) **(B)**. On hospital day 7, a brain MRI was done again and showed acute multiple cerebral infarcts in the left cerebral hemisphere **(C,D)**. On hospital day 25, cranial CT revealed significantly increased low-density shadow in the left frontal, temporal, and parietal lobes **(E–G)**. The MRI **(H,I)** and MRA **(J)** re-examination demonstrated that the lesions in the left cerebral hemisphere were increased significantly, and a cerebral aneurysm formed in the intracavernous segment of the left internal carotid artery (ICA). On hospital day 29, a repeat head MRI showed markedly enlarged size of the left temporal lesions with abscess formation and bleeding **(K,L)**. On hospital day 37, brain MRI showed a decreased size of the left temporal lesions but with perifocal evident edema **(M)**. On hospital day 55, a repeat head MRI showed that the lesions and edema of the left temporal lobe was improved, but hydrocephalus became even worse **(N,O)**. On hospital day 58, cranial CT showed intraventricular extension and hydrocephalus **(P)**.

On admission, physical examination showed poor mental status and subtle abduction limitation in the left eye without other neurological signs or symptoms. The patient underwent a lumbar puncture on the day of admission. Cerebrovascular fluid (CSF) showed a normal WBC count (4/μl, reference range 0–8/μl), mildly increased protein level (0.6 g/L, reference range 0.15–0.45 g/L), reduced chloride levels (114.6 mmol/L, reference range 118–132 mmol/L), and normal glucose levels (3.97 mmol/L, reference range 2.5–4.2 mmol/L; Table 1). Pre-antibiotic blood culture was taken with a negative result. Additional laboratory examinations, such as serology testing for HIV, hepatitis B/C, syphilis, rheumatic diseases, and tumor markers, were normal. Cranial magnetic resonance imaging (MRI) was normal (Figure 1A), and there was no evidence of intracranial aneurysm on brain magnetic resonance angiography (MRA; Figure 1B). The diagnosis of viral encephalitis was considered and treatment with intravenous acyclovir (10 mg/kg) was initiated and continued every 8 h. However, with frequent low fever, the illness was not well-controlled. Considering the likelihood of bacterial infections, ceftazidime (2 g every 12 h) was given on the third day of admission. On hospital day 7, the patient developed significant diplopia and weakness in the right upper extremity with a brain MRI showing acute multiple cerebral infarcts in the left cerebral hemisphere (Figures 1C,D). On the same day, the patient underwent a lumbar puncture once more, and the macroscopic appearance of the CSF was slightly yellow with a pressure of 190 mmH_2_O (normal range, 80–180 mmH_2_O). CSF results are shown in Table 1. The patient was diagnosed with intracranial infection and transferred to the Department of Neurology.

**Table 1:** Dynamic cerebrovascular fluid (CSF) analysis throughout hospitalization.

**CSF-Variable**	**Day 1**	**Day 7**	**Day 25**	**Day 28**	**Day 58**	**Day 63**
CSF opening pressure (cm of water)	17.5	19	12	13.5	22	13
General appearance	Colorless	Slightly yellow	Colorless	Colorless	Colorless	Colorless
White cell count (10^6^/L)	4	238	300	50	80	8
Mononuclear cells percentage (%)	–	42	63	94	–	–
Protein (g/L)	0.6	1.27	1.33	0.76	2.01	0.32
Glucose (mmol/L)	3.97	3.18	3.58	2.94	2.01	4.01
Chloride (mmol/L)	114.6	114.8	120.7	114.9	113.7	119
Microbiological testing	–	–	–	–	–	–

Neurological examination showed drowsiness, left abducens nerve palsy, degree 4 muscle strength in right upper limb, and neck rigidity (+). The culture of bacteria and mycobacterium tuberculosis and the detection of viral nucleic acid for retrovirus, herpes virus 1 and 2, rhinovirus, Epstein-Barr (EB) virus, and cytomegalovirus in CSF were negative. Additionally, the brucellosis agglutination test, Japanese encephalitis virus antibodies detection, T cell spot test for tuberculosis (T-SPOT), tuberculin test, and mycobacterium tuberculosis/rifampicin resistance test (X-pert) were also negative. Since our patient had such signs, the diagnosis of tuberculous meningitis was considered. The patient was administered an anti-tuberculous therapy that included isoniazid (0.6 g once daily), rifampicin (0.45 g once daily), ethambutol (0.75 g once daily), and pyrazinamide (0.5 g three times daily). In the following days, the body temperature returned to normal, but the patient still had intermittent headache. Compound codeine phosphate and ibuprofen sustained release tablet (0.2 g/13 mg two times daily) were added to control the headache. On hospital day 16, the patient developed frequent nausea and vomiting. The mental state of the patient also became very poor yet no fever, abdominal pain, or blurred vision were observed. Physical examinations, laboratory tests, and abdominal computed tomography (CT) scans could not reveal the cause of the patient's deterioration. We suggested that it might be the side effects of anti-tuberculosis drugs. Ondansetron (8 mg) and metoclopramide (10 mg) were intravenously utilized for anti-emesis. His gastrointestinal symptoms were improved. However, the mental state was getting even worse. On hospital day 25, the patient was presented with near-complete left oculomotor nerve palsy with blepharoptosis and dilated pupils on the left side. CSF results showed an increased WBC count (300 × 10^6^/L) with 63% mononuclear cells (Table 1). Cranial CT revealed significantly increased low-density shadow in the left frontal, temporal, and parietal lobes (Figures 1E–G). As a result of the disease progression, the patient was transferred to a superior hospital.

Detailed neurological examinations and evaluations were performed on this patient again. The MRI (Figures 1H,I) and MRA (Figure 1J) re-examination demonstrated that the lesions in the left cerebral hemisphere were increased significantly, and a cerebral aneurysm was formed in the intracavernous segment of the left internal carotid artery (ICA). In addition, the sphenoid sinus mucosa was thickened. Taking the possibility of bacterial infection into consideration, the patient was treated with ceftriaxone (2 g every 12 h). On hospital day 28, the patient underwent a lumbar puncture examination again. CSF results are shown in Table 1. Metagenomic sequencing of viral and bacterial genomes from this patient's CSF sample was performed with a negative result. Unfortunately, the patient fell into a coma the following day and was admitted to the intensive care unit (ICU). Head MRI showed a markedly enlarged size of the left temporal lesions with abscess formation and bleeding, accompanied by peripheral ring-shaped enhancement (Figures 1K,L). Due to the patient‘s poor condition, the anti-infection regimen was adjusted to an intravenous drip of biapenem (0.3 g every 12 h) and vancomycin (1 g every 12 h). The patient was gradually recovered consciousness within the next 7 days. On hospital day 37, brain MRI showed a decreased size of the left temporal lesions but with perifocal evident edema (Figure 1M). To further clarify the nature of the intracranial lesions, the patient was transferred to the Neurosurgical Department to perform the needle biopsy with the assistance of real-time neuro-navigation on hospital day 38. The next day, this patient underwent the procedure, and the surgical specimen was sent for smear, culture, and deoxyribonucleic acid sequencing. According to the sequencing results, the patient was diagnosed with bacterial meningitis caused by a mixed infection of oral microflora dominated by anaerobes, such as *Fusobacterium nucleatum* (69.79% relative abundance), *Prevotella intermedia* (20.77% relative abundance), *Bacteroides uniformis* (1.35% relative abundance), *Campylobacter rectus* (1.26% relative abundance), *Oral peptostreptococcus* (0.08% relative abundance), *Streptococcus constellatus* (0.02% relative abundance), *Torque teno* virus 15, and *Treponema socranskii*. The anti-microbial regimen (biapenem combined with vancomycin), which could sufficiently cover anaerobe, was used continuously. For the poor oral hygiene, the patient received a detailed oral examination and nursing intervention. In the following days, the overall condition of the patient was significantly improved, but there was still a loss of appetite. However, the clinical symptoms worsened from the hospital day 53. The patient was presented with severe lethargy, headache, nausea, and vomiting. Repeat brain MRI showed that the lesions and edema of the left temporal lobe were improved, but hydrocephalus became even worse (Figures 1N,O). The patient was transferred to the Department of Neurosurgery of our hospital at the insistence of family of the patient on hospital day 58.

Neurological examination revealed consciousness disturbance with a Glasgow Coma Scale (GCS) of 9 (E2V2M5), right hemiplegia, left eyeball fixation, pupil dilation with ptosis on the left side, and stiffness in the neck. Cranial CT showed intraventricular extension and hydrocephalus (Figure 1P). We considered that the aggravation of this patient's disease might be related to the progressive hydrocephalus. Then, the patient underwent ventriculostomy on hospital day 59. The anti-infection therapy regimen previously described was changed to the combination of meropenem (2 g every 8 h) and linezolid (0.6 g every 12 h). The clinical symptoms of the patient were improved significantly, and the patient gradually regain consciousness. On hospital day 85, this patient underwent a ventriculoperitoneal shunt operation, and then the mental state of the patient improved further. On hospital day 97, when the patient was discharged, patient was able to walk with assistance and engage in simple conversation but showed a loss of appetite and significant weight loss.

**Figure 2:**
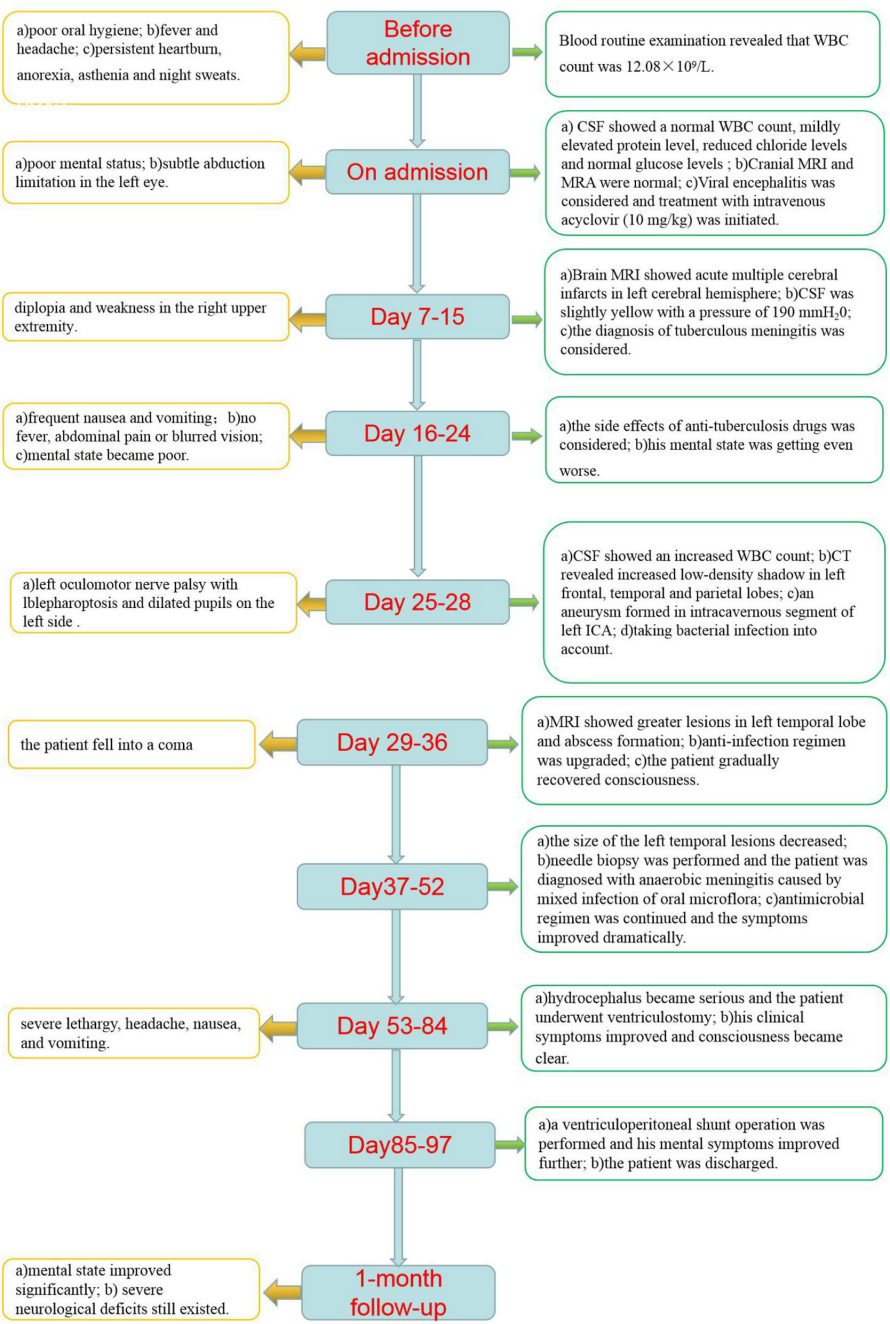
Clinical course of the case.

In the 1-month follow-up, the patient could walk without the assistance of a cane or brace. However, severe neurological deficits, such as diplopia and right hemiplegia, still existed. To prevent disease recurrence, we suggested that the patient should maintain good oral hygiene and regular follow-up at the stomatology clinic. Because of the high risk of rupture and mortality of the patient's intracranial aneurysm, surgical treatment was advised. However, the patient refused further examination and treatment for financial reasons. Regular follow-ups will be carried out for our patients. The clinical course is summarized in Figure 2.

## Discussion

Adult bacterial meningitis (ABM) caused by anaerobic bacteria infection is a rare condition and is often overlooked or under-diagnosed (5–7). The clinical outcome is usually more severe. With mastoiditis and acute or chronic otitis media being the most common triggers, other triggers include oropharyngeal infection, sinusitis, craniotomy, poor oral hygiene, head trauma, tumors of the head and neck regions, and sacrococcygeal dermal sinus (2, 8–16). Our patient was presented with fever and headache as the initial symptoms and then developed left ophthalmoplegia, right hemiplegia, and disturbance of consciousness. Brain imaging showed that cerebral infarcts, brain abscesses, intracranial aneurysm, and severe hydrocephalus were appeared gradually. CSF showed a significantly increased WBC count. Eventually, the diagnosis of anaerobic meningitis was made by deoxyribonucleic acid sequencing from the brain abscess pus. According to the sequencing results, this was a mixed infection of various oral microorganisms, mostly anaerobic bacteria. These bacteria are commonly found in the mucosal surfaces of the mouth and upper respiratory tract. As our patient long had poor oral hygiene, we speculated that chronic oral inflammations might be the predisposing factor of anaerobic meningitis in this patient. Oral flora can theoretically enter the brain through the following ways (3): (a) systemic hematogenous bacteremia; (b) direct venous drainage *via* the two main venous networks leading to the cavernous sinus, the facial, and the pterygoid vein systems; (c) inoculation *via* contiguous extension or by the introduction of foreign objects; and (d) lymphatic drainage. Our patient had no symptoms of bacteremia, the results of blood cultures and echocardiography were all negative. The D-dimer test was within normal ranges. Brain imaging showed that intracranial lesions, such as cerebral infarcts, brain abscess, and intracranial aneurysm, were all on the left side of the cerebral hemisphere. Based on this, we excluded systemic hematogenous bacteremia diagnosis. Although cranial imaging showed sphenoid sinusitis, we saw no evidence of osseous destruction. From the considerations above, we thought that the most likely route of infection in our patient was hematogenous dissemination *via* venous pathways. The spread of bacteria through the blood led to the left cavernous sinus involvement, which resulted in near-complete left oculomotor nerve palsy with blepharoptosis and dilated pupils on the left side (palsy of the oculomotor, trochlear, or abducens cranial nerves). A possible explanation for why all the intracranial lesions occurred in the left cerebral hemisphere was that the left implantation of mandibular dentures could increase the probability of the destruction of the anatomical barrier of the left oral mucosa.

Bacterial aneurysms, also known as infective or microbial aneurysms, are rare inflammatory neurovascular lesions that account for 0.7–6.5% of all intracranial aneurysms (17, 18), most of which are secondary to infectious endocarditis (19–21). They often exhibited multiple lesions, hemorrhagic presentation, and septic cerebral embolism (22–24). Among these, septic embolism was likely the cause of multiple cerebral infarcts in our patient. Currently, there are two hypotheses that explain the pathogenesis of bacterial aneurysms that include the vasa vasorum hypothesis or embolic (25–28). The former is the most widely accepted mechanism of pathogenesis. It proposes that bacteria from septic emboli escape through the vasa vasorum and cause severe inflammation of the adventitia. The infection then spreads inwardly. The arterial pulsation against the weakened vessel wall eventually results in aneurysm formation and enlargement. The embolic hypothesis posits the centrifugal spread of inflammation and proposes that septic emboli occlude the arterial lumen and that destruction spreads outward from the intima to the adventitia (28). During the initial admission, there were no intracranial aneurysms observed on cranial MRA for our patient. With the progression of the disease, a cerebral aneurysm of the intracavernous segment in the left ICA was discovered on hospital day 25. We considered that this aneurysm was occurred according to the vasa vasorum theory *via* cavernous sinus phlebitis. The possible mechanisms of oral pathogens affecting inflammatory remodeling in the wall of the intracranial aneurysm are macrophage infiltration, activation of toll-like receptors by lipopolysaccharide (LPS), activation of the complement system, endothelial dysfunction, and so on (29). So far, bacterial intracranial aneurysms caused by oral anaerobes are very rare. At present, only two cases were reported. Park et al. (9) reported a case of intracranial mycotic aneurysm caused by *P. intermedia* associated with chronic sinusitis and successfully identified the bacteria by 16S rRNA sequencing. In Kyoya's case (30), a 62-year-old man displayed multiple infectious intracranial aneurysms, intracerebral hemorrhage, and epistaxis after tooth extraction. By culturing the aspirate of the abscess, the pathogenic bacterium was ultimately identified as oral bacteria, such as Gram-negative anaerobic rods (*Fusobacterium sp*.) and Gram-positive anaerobic cocci (*Parvimonas micra*). Combined with appropriate anti-infective treatment and surgical intervention, the patient finally achieved a good result. A previous study found that earlier and adequate antibiotic therapy may have increased the chance of improved aneurysmal outcomes (31). The aneurysms with a high risk of rupture, such as saccular morphology, may correlate with unfavorable aneurysmal outcomes, which need to be treated more aggressively than with antibiotics alone (31, 32). After effective anti-anaerobes treatment, there was no further enlargement or rupture of the intracranial aneurysm in our patient based on the subsequent re-examination. However, the patient refused further surgical intervention for financial reasons.

According to the sequencing results from the brain abscess pus, anaerobes associated with intracranial infection in our patient included *F. nucleatum, P. intermedia, B. uniformis*, and *O. peptostreptococcus*. *F. nucleatum* is a Gram-negative anaerobic bacillus with species-specific reservoirs in the human mouth, gastrointestinal tract, and elsewhere. According to some studies, this bacterial is associated with meningitis or related complications that include acute cerebral infarction, brain abscess, cerebral sinus vein thrombosis (CSVT), and hydrocephalus (5, 33, 34). *F. nucleatum* is often found to be involved in polymicrobial infections (4). In a case series of five Fusobacterial brain abscesses, only one patient had a monomicrobic *F. necrophorum* infection, whereas all others had polymicrobic infections involving *F. nucleatum* (35). The authors suggested that the less virulent *F. nucleatum* requires synergy from other organisms (35). Prevotella is the second most abundant genus. Several studies have reported that Prevotella was associated with meningitis and was involved in the formation of brain abscesses (3, 5, 9). In Mo's case, a 48-year-old man, with mixed infections caused by *P. intermedia* and *S. constellatus*, presented a large area of ischemic infarction and ultimately abandoned the treatment due to a worsening condition (36). *B. uniformis* is also a strict anaerobe, Gram-negative bacillus, and part of the normal oral cavity and gastrointestinal flora. Maj et al. (37) found that this bacterium was involved in the formation of brain abscesses as part of a mixed infection of multiple microorganisms. Moreover, some case reports or case series showed that *O. peptostreptococcus* was associated with intracranial infection or related complications (3). In the initial stage of the disease, although Ceftazidime was used to treat bacterial infections, a pitfall of this regimen was the incomplete coverage of the above anaerobes, which led to the fast progression of infection. After using a combination of biapenem and vancomycin, the patient's infection was effectively controlled. Currently, there is no consensus as to the best regimen for the anti-infection treatment of anaerobic meningitis, further study is still in need. The timely use of broad-spectrum antibiotics covering above different anaerobic flora is essential to improving the prognosis of the disease. The sensitivity and resistance to the drug and the difference in blood-brain barrier penetration of the anaerobic bacteria species should be considered comprehensively when selecting an appropriate antibiotic. Especially, when there is evidence that the infection may originate from oral flora, physicians should strongly consider requesting anaerobic culture and altering patient treatment to one that includes anaerobic anti-microbial coverage.

Anaerobic bacteria are usually difficult to detect, especially after the initiation of anti-microbial therapy (7). Alternatively, clinicians rarely use a scheduled specific anaerobic bacteria culture of CSF specimens in clinical practice (7, 38). All these factors made the diagnosis of anaerobic meningitis very difficult. When culture methods fail to yield an etiological answer, further microbiological evaluation is justified. Suitable molecular biologic approaches, such as high-throughput sequencing, may help to provide a diagnosis.

## Conclusion

In summary, anaerobic meningitis caused by a mixed infection of oral microflora is rare, especially when complicated with an infected intracranial aneurysm, which is usually associated with underlying causes. This report illustrates the importance of considering oral anaerobes as a cause of meningitis and intracranial aneurysm. It underlines the usefulness of molecular techniques in the diagnosis of infections with anaerobic bacteria and the importance of early intervention for a good prognosis.

## Data availability statement

The datasets presented in this article are not readily available because of ethical and privacy restrictions. Requests to access the datasets should be directed to the corresponding author/s.

## Ethics statement

The studies involving human participants were reviewed and approved by Jincheng People's Hospital Affiliated to Shanxi Medical University, Jincheng, China. The patients/participants provided their written informed consent to participate in this study.

## Author contributions

LJ and LX put forward research ideas. FY and CL took the responsibility of communicating with the patient's family and obtaining the authorization in this paper. HC and DZ responsible for drafting articles. All authors contributed to the article and approved the submitted version.

## Conflict of interest

The authors declare that the research was conducted in the absence of any commercial or financial relationships that could be construed as a potential conflict of interest.

## Publisher's note

All claims expressed in this article are solely those of the authors and do not necessarily represent those of their affiliated organizations, or those of the publisher, the editors and the reviewers. Any product that may be evaluated in this article, or claim that may be made by its manufacturer, is not guaranteed or endorsed by the publisher.
